# Effects of Hyperlipidemia on Osseointegration of Dental Implants and Its Strategies

**DOI:** 10.3390/jfb14040194

**Published:** 2023-03-30

**Authors:** Haiyang Sun, Shuhuai Meng, Junyu Chen, Qianbing Wan

**Affiliations:** State Key Laboratory of Oral Diseases, National Clinical Research Center for Oral Diseases, Department of Prosthodontics, West China Hospital of Stomatology, Sichuan University, Chengdu 610041, China

**Keywords:** hyperlipidemia, dental implantation, osseointegration, surface modification, statins

## Abstract

Hyperlipidemia refers to the abnormal increase in plasma lipid level exceeding the normal range. At present, a large number of patients require dental implantation. However, hyperlipidemia affects bone metabolism, promotes bone loss, and inhibits the osseointegration of dental implants through the mutual regulation of adipocytes, osteoblasts, and osteoclasts. This review summarized the effects of hyperlipidemia on dental implants and addressed the potential strategies of dental implants to promote osseointegration in a hyperlipidemic environment and to improve the success rate of dental implants in patients with hyperlipidemia. We summarized topical drug delivery methods to solve the interference of hyperlipidemia in osseointegration, which were local drug injection, implant surface modification and bone-grafting material modification. Statins are the most effective drugs in the treatment of hyperlipidemia, and they also encourage bone formation. Statins have been used in these three methods and have been found to be positive in promoting osseointegration. Directly coating simvastatin on the rough surface of the implant can effectively promote osseointegration of the implant in a hyperlipidemic environment. However, the delivery method of this drug is not efficient. Recently, a variety of efficient methods of simvastatin delivery, such as hydrogels and nanoparticles, have been developed to boost bone formation, but few of them were applied to dental implants. Applicating these drug delivery systems using the three aforementioned ways, according to the mechanical and biological properties of materials, could be promising ways to promote osseointegration under hyperlipidemic conditions. However, more research is needed to confirm.

## 1. Introduction

Hyperlipidemia is a disease in which the level of plasma lipids (including cholesterol, triglycerides, and lipids) abnormally increases and exceeds the normal range. Sometimes, some lipoprotein levels increase in the hyperlipidemic plasma, such as high-density lipoprotein. Therefore, some researchers claim that hyperlipidemia should be renamed dyslipidemia [1]. In this review, the range of hyperlipidemia includes abnormalities of cholesterol, triglycerides, and lipoprotein. At present, the population with hyperlipidemia is very large. In the United States, the administration of statins is recommended in approximately 5.6 million (48.6%) adults over the age of 40 for the treatment of hyperlipidemia, and 21% of children and adolescents aged 6–19 have at least one abnormal blood lipid parameter [2]. In China, the overall prevalence of hyperlipidemia among adults is 40.40%.

In China, the average number of teeth loss by the elderly aged over 60 is 10.7, and the number of teeth lost by the elderly is as high as 80%. It is estimated that there are about 250 million patients with tooth loss, and the number of teeth loss increases by at least 5% every year. The population of patients with hyperlipidemia who lose teeth is very large. Dental implant is an effective, comfortable and widely used method for replacing missing teeth by stably connecting the implant with the jawbone and then installing the crown on the abutment. The stability of the implant and bone connection is very important for the life of dental implants. As early as 1962, Branemark accidentally discovered the strong combination of titanium metal and bone and applied titanium to the study of dental implants. In 1977, he proposed the theory of osseointegration of implants and bone tissue [3]. Osseointegration of an implant is defined as the direct structural and functional integration of the implant surface with the surrounding well-developed bone tissue without fibrous tissue ingrowth at the interface [4]. This theory has always been considered as the theoretical basis of modern oral implantology, and osseointegration is also recognized as the most ideal combination of implants and surrounding bone tissue. Thus far, there have been various studies on dental implant materials aimed at promoting osseointegration, improving the stability of the implant, and prolonging the life of the implant. However, hyperlipidemia can affect bone metabolism and increase the incidence of osteoporosis [5].

Implant modification is one of the main ways to promote the success rate of osseointegration and implantation under special pathological conditions; currently, good mechanical and biological effects can be obtained by loading different bioactive molecules or drug coatings on the implant surface [6,7]. However, a review regarding implant modification strategies in the hyperlipidemic environment has not been systematically performed in recent years. In this review, we searched MEDLINE, EMBASE for studies published between 1985 and 2023 with the terms “hyperlipidemia”, “dyslipidemia”, “cholesterol”, “obesity”, “osseointegration”, gaining more insights into the effects and mechanism of hyperlipidemia on osseointegration. Furthermore, we proposed the potential effective strategies of the implants in the hyperlipidemic environment, providing a basis for the clinical application of these improved measures.

## 2. Implants in Hyperlipidemia

Several experiments have investigated the status of dental implants placed on long bones in hyperlipidemic animal models, and the results have shown that hyperlipidemia significantly affects implant osseointegration [8,9,10,11,12]. The influence of hyperlipidemia on the osseointegration of titanium implants is manifested in many aspects, and the internal mechanism is complex.

### 2.1. Negative Effects of Titanium on the Osseointegration under Hyperlipidemic Conditions

The titanium implant itself does not favor osseointegration in a hyperlipidemic environment. Hu et al. found that the titanium oxide layer on the surface of titanium significantly increases the level of reactive oxygen species (ROS) in mice [13]. Titanium oxidizes low-density lipoproteins in the blood to a certain extent to form oxidized low-density lipoprotein [14]. When titanium implants are placed in patients with hyperlipidemia, the produced oxidized lipids enhance the activity of osteoclasts, promote bone resorption, and induce local inflammation as well as oxidative stress [15], which are harmful to osseointegration.

### 2.2. Effects of Hyperlipidemia on Bone Tissue around Implants

The results of an experiment on New Zealand rabbits showed that hyperlipidemia has a negative impact on implant stability [8]. Some researchers also performed experiments on mice, and the results suggested that high blood lipids do not favor implant osseointegration [9,16]. It was further proposed that poor osseointegration may be related to changes in morphology and mechanical properties caused by decreased bone density and mineral content [10]. The bone–implant connection in rats with high blood lipids does not change significantly, but the bone filling rate around the implant decreases [11]; similar conclusions were drawn in rabbits [12].

The abnormal levels of cholesterol and other lipids under hyperlipidemia affect the differentiation and activation of osteoblasts or osteoclasts, which are not conducive to bone metabolism [17]. Patients with hyperlipidemia may have lower bone mineral density and bone mineral content than patients with normal blood lipids. The results of an epidemiological survey showed that hyperlipidemia is associated with osteoporosis [18]. Osteoporosis can affect the jaw, consequently affecting the bone density and bone mineral content of the jaw [19]. Osteoporosis is also a potential risk factor for implant surgery [20]. Therefore, osteoporosis caused by hyperlipidemia is also an important factor affecting osseointegration.

**Figure 1:**
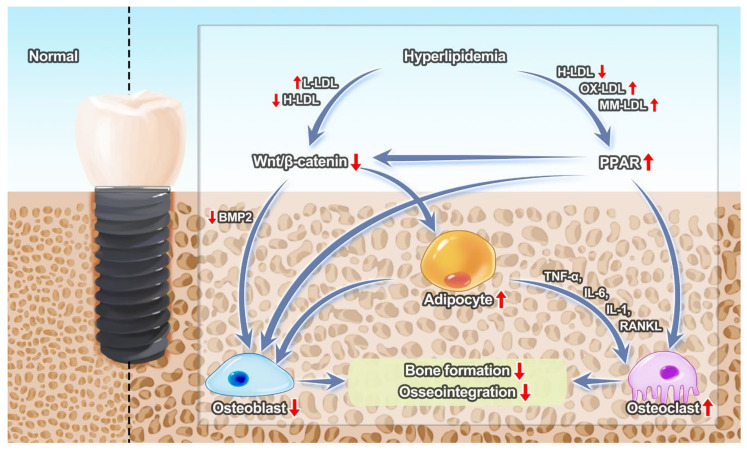
Inhibition mechanism of osseointegration under hyperlipidemia. L-LDL, low-density lipoprotein; H-LDL, high-density lipoprotein; OX-LDL, oxidized low-density lipoprotein; MM-LDL, minimally modified low-density lipoprotein.

Some researchers have further explored the mechanism by which hyperlipidemia affects implant osseointegration (Figure 1). Studies have shown that osteoblasts and trabecular bone decrease and osteoclasts increase in rats with hyperlipidemia.

Bone marrow mesenchymal stem cells have the ability to differentiate into adipocytes and osteoblasts. The Wnt/β-catenin signaling pathway is inhibited by the increased low-density lipoprotein and reduced high-density lipoprotein in the hyperlipidemic environment [21]. Subsequently, bone marrow mesenchymal stem cells differentiate mainly into adipocytes, while osteoblast differentiation is inhibited and bone formation is reduced through the bone morphogenetic protein 2 (BMP2)-Wnt/β-catenin [22,23,24]. The PPARγ in the PPAR signaling pathway is upregulated by the change of lipid and lipoprotein levels in the hyperlipidemic environment, such as by high-density lipoprotein, oxidized low-density lipoprotein and minimally oxidized low-density lipoprotein. The PPAR signaling pathway inhibits the expression of osteoblast transcription factors, such as Runx2 and Osx, thereby inhibiting the differentiation of mesenchymal stem cells into osteoblasts [25]. The PPAR signaling pathway promotes the differentiation of osteoclasts and leads to bone loss. In addition, it inhibits the Wnt/β-catenin signaling pathway [26]. Adipocytes themselves inhibit osteoblasts [27] and secrete factors such as TNF-a, IL-1, IL-6 and RANKL to promote osteoclast differentiation [28,29,30,31].

### 2.3. Effects of Hyperlipidemia on Inflammation about Implants

Hyperlipidemia aggravates the inflammatory process by stimulating the expression of pro-inflammatory cytokines and by boosting ROS production. It increases the susceptibility of the body to periodontitis and peri-implantitis [32,33]. Increased lipids in patients with hyperlipidemia lead to the upregulation of various inflammatory factors, induce systemic or local inflammatory responses, and seriously affect the survival rate of implants. The permeability of the vascular endothelium and basement membrane is risen and the exudation of lymphocytes and plasma cells is increased in gingiva, which reveal that the establishment of hyperlipidemia may also induce the destruction of periodontal tissue [34]. The research results of Cutler et al. showed that there is a significant relationship between periodontal inflammation and hyperlipidemia, and elevated triglycerides could regulate the production of IL-1β stimulated by Porphyromonas gingivalis [35]. A previous study has suggested that the accumulation of ROS induced by hyperlipidemia leads to osteoblastic dysfunction and implant osseointegration dysfunction [16]. Meanwhile, in hyperlipidemic environments, triglyceride-rich lipoproteins can increase the expression of inflammatory promoting factors such as ICAM-1, VCAM-1, and IL-6, causing oxidative stress, thereby promoting neutrophil aggregation [36]. Excess cholesterol induces the release of inflammatory cytokines such as IL-6, IL-10 by activating the NF-κB signaling pathway [37]. The accumulation of cholesterol in macrophages can also change the ratio of M1/M2 macrophages, promote the M1 proinflammatory environment, and thereby increase the number of monocytes/macrophages in the circulation [38]. These factors lead to a series of symptoms such as peri-implant bone resorption, loss of the implant–bone interface, and peri-implant pocket formation. Hyperlipidemia affects the function of neutrophils, leading to early wound healing after implant surgery. Moreover, the dysfunction of neutrophils leads to acute periodontitis, which does not favor the stability of implants.

## 3. Local Drugs Injection

There are non-drug methods used to treat hyperlipidemia, such as diet control and weight loss [39]. In this review, we were only concerned with drug methods. Bile acid-binding resins, niacin, fibrates and statins are the most commonly used lipid-regulating drugs [40]. By searching in MEDLINE and Web of Science for these drugs and osseointegration, we found that only statins were injected around implants and promoted osseointegration.

Statins are HMG-CoA reductase inhibitors, which are the most effective drugs in the treatment of hyperlipidemia [41]. In addition to the blood lipid regulating effect, they have a variety of other effects, including the regulation of bone metabolism. Statins inhibit the synthesis of farnesyl pyrophosphate, reduce cell cholesterol, activate the Ras-PI3KAkt/MAPK signaling pathway, upregulate the expression of BMP-2 and Runx2 and promote osteogenesis. In addition, they inhibit osteoblast apoptosis through the TGFβ/Smad3 pathway and prevent osteoclastogenesis through the OPG/RANKL/RANK pathway [42]. In general, statins prevent osteoporosis [43], consequently preventing tooth loss [44]. From this point of view, statins have considerable application prospects in patients with hyperlipidemia and are beneficial in the improvement of bone mass in patients with hyperlipidemia.

Simvastatin is a frequently used statin included in various drug delivery systems and is widely used in the treatment of cardiovascular diseases [45,46], tumors [47] and Alzheimer’s disease [48]. Dental implant osseointegration is a specific phenomenon of osteogenesis, and the promotion of osteogenesis can effectively promote dental implant osseointegration. Simvastatin has been widely used to promote implant osseointegration and bone regeneration. Vascular growth factor (VEGF) plays an important role in the growth of blood vessels in bone tissue and the development of bone tissue [49]. Studies have found that simvastatin promotes the growth and development of bone cells by regulating the expression of VEGF [50]. It was also demonstrated that simvastatin not only promotes the development of osteoblasts but also inhibits the formation of osteoclasts by inhibiting the production of cholesterol on the osteoclast cell membrane [51].

Previous studies have shown that the systemic use of statins is beneficial to implant osseointegration even under hyperlipidemic conditions [52,53,54,55]. However, the bioavailability of statins in bone tissue is low, which means that the systemic use of statins requires administration of large doses, thus enhancing side effects [43]. Therefore, the effective local delivery of statins to the peri-implant bone tissue has become a new focus.

Injecting statins locally is the simplest way (Table 1) [52,53,54]. Local injection of simvastatin was found to be beneficial to osseointegration [56]. Moriyama et al. injected fluvastatin with propylene glycol alginate (PGA) gel into the bone of rats before implanting titanium implants and found that the histomorphological and mechanical properties of peri-implant bone in the fluvastatin group were superior to those in the non-fluvastatin group, and the push-in strength was higher [57,58]. Injecting poly (lactic-co-glycolic acid) (PLGA) microspheres containing fluvastatin could also promote osseointegration and increase the mechanical properties of bone [59]. Although not all statins have been applied to dental implants under hyperlipidemic conditions, we speculate that they could be highly effective.

Some researchers have injected enhancers or inhibitors locally by genetic engineering. Ren et al. used microarray analysis to study the effects of hyperlipidemia on osseointegration and found that applying Sdccag3-enhancer, lncRNA-MSTRG.97162.4-enhancer and miR-193a-3p-inhibitor could boost bone formation of BMSC in vitro and improve osseointegration in vivo [60]. MiR-29a-3p-enhancer was also beneficial in implant osseointegration [61]. Although such modification had a clear goal and obvious effect, it was difficult to apply to clinical practice considering ethical issues. We still need to find an easy and fast way to improve osseointegration and bone formation.

Local drug injection is not an optimal method under a hyperlipidemic environment. On the one hand, it is difficult to evenly fill the implant cavity with drugs. On the other hand, it is also not possible to ensure that the drugs remain on the surface of the implant after rinsing and blood flow. In addition, evaluating the osseointegration effect of implants mainly relies on bone–implant contact analysis, peri-implant bone volume analysis, and push-out experiments. Thus, it is difficult to accurately detect the osseointegration of each fine area of the implant. When the drug distribution is uneven after local injection, the experimental results of osseointegration will not be accurate. Therefore, implant modification aiming at evenly covering the implant surface with drugs is a more efficient and reliable method for the implant under a hyperlipidemic environment.

## 4. Implant Surface Modification under Hyperlipidemic Conditions

As mentioned above, titanium is harmful to implant osseointegration under hyperlipidemia. Some studies have optimized implant materials, which include titanium alloy implants and zirconia implants. However, changing the materials of implants cannot maintain the advantages of the good mechanical properties of titanium [62]. Therefore, the surface modification of the titanium implant is a method that not only retains the good mechanical properties of the titanium metal, but also optimizes the osseointegration of the implant. This aspect has always been the top priority of implant research. There are various ways to modify the surface of implants, such as acid etching and grit blasting, both promoting osseointegration by properly improving the surface roughness of the implants [63]. Another surface modification of implants is coating. Good mechanical and biological effects can be obtained through different bioactive molecules or drug layers [7]. Coating on the rough surface is the most common method of modification.

This review summarized the strategies of implant surface modification that have been promising for hyperlipidemia in recent years (Table 2).

### 4.1. Statin-Based Implant Surface Modification

Surface modification is an effective method used to deliver statins [57]. Most researchers have previously opted for a direct coating on the roughened implant surface. A micro-arc oxidation-treated roughened surface loaded with simvastatin showed a significant effect in promoting osseointegration [64], and this promoting effect also exists in the case of low bone mass [65,66]. Lopez-Alvarez M et al. covered mesoporous TiO_2_ with simvastatin to modify the surface of implants and further found that the mesoporous surface promotes the loading of simvastatin as well as osseointegration [67]. A biomimetic calcium phosphate coating was also used for the local delivery of simvastatin and exhibited good osseointegration effects [68]. The existing methods of local delivery of statins applied to dental implant osseointegration are mainly used to roughen the surface of the implant and to place it in a statin solution. However, the drug loading efficiency is low; thus, there is still the need to explore more efficient delivery routes. Pullisaar et al. selected alginate hydrogel as a container to deliver simvastatin and found that this coating released simvastatin progressively and sustainedly [69]. Nanohydroxyapatite could also be a carrier for simvastatin, and they work together to promote bone formation [70]. Placing statins into a drug delivery system for coating will achieve a longer release time and better effect than a direct coating.

### 4.2. Vitamin D Based Implant Modification

Vitamin D deeply affects calcium and phosphorus metabolism, as well as bone remodeling. Serious deficiency of vitamin D leads to rickets in children and osteomalacia in adults. Low deficiency of vitamin D also has harmful effects on bone tissue, which is not able to osseointegrate around implants [72]. 1,25 (OH)2 vitamin D3 is an effective promoter of osteoblast–osteocyte transformation [73]. Current research believes that vitamin D reduces bone resorption, improves bone structure, and inhibits osteoclastogenesis by activating T cell factors [74]. Previous studies have found that vitamin D metabolites increase serum osteocalcin levels in animals at risk of abnormal lipid metabolism, leading to new bone formation in the trabecular bone and cortical bone, effectively inhibiting bone resorption [75,76]. Moreover, a vitamin D coating on the surface of the implant reduces alveolar bone loss in hyperlipidemia, although this promotion effect is very limited [71]. Therefore, it is speculated that the use of vitamin D in hyperlipidemic patients with low vitamin D levels may lead to good results. Vitamin D is also closely related to lipid levels, especially cholesterol [77,78]. However, the used of vitamin D still needs further exploration using in vitro and in vivo experiments.

Implant surface modification is an ideal method used for promoting osseointegration, especially when combined with sustained and controlled-release drug delivery systems. It is expected that the implant and bone should eventually form to a rigid interface; thus, the active agents/drugs should be tightly adhered to the implant surface during implant modification. The common methods of implant modification are to load drug delivery systems onto the porous implant surface or to form chemical bonds between the drugs and the rough and active implant surface. Among the current commercial implant modification technologies, anodic oxidation can form titanium dioxide mesopores on the titanium surface. Alkali-heat treatment and sandblasting acid etching techniques can also endow the implant surface with rich chemical bonds, which would be suitable for further integration with drug delivery systems. Therefore, to solve the interference of hyperlipidemia in osseointegration, implant modification with targeted drugs requires not only efficient drug delivery systems but also active implant surfaces to provide ideal reaction regions for drugs.

## 5. Bone Grafting Material Modification

Other than local drug injection and implant surface modification, modifying the bone grafting materials can also encourage osseointegration. Mansour et al. formulated simvastatin as granules in hydroxypropyl methyl cellulose, which was used as a bone grafting material and promoted bone healing and osseointegration [79]. In another bone grafting material, simvastatin was encapsulated by poly(lactic acid-co-glycolic acid)-polyethylene glycol (PLGA-PEG) nanoparticles loaded within a bioceramic scaffold [80]. Simvastatin was also added to methylcellulose gel as a bone grafting material [81].

Due to the inherent porosity of certain bone grafting materials, they can easily encapsulate targeted drugs for hyperlipidemia treatment. Loading drugs into bone grafting materials might be an alternative way to promote osseointegration in hyperlipidemic environments. However, because the bone grafting material does not completely wrap the implant, but only covers the upper bone defect sites, this largely limits the drug’s osseointegration-promoting effect. Therefore, loading drugs with bone grafts is not an optimal approach from our perspective.

## 6. Promising Strategies for Promoting Implant Osseointegration

### 6.1. The Application of Statins

Many statin delivery systems related to bone regeneration that also use simvastatin as a component are available [82]. However, only a few of them have been used in dental implantation. In Table 3, we list the articles on statin delivery systems used for bone formation, which are promising in promoting implant osseointegration. Considering the therapeutic effects of statins on hyperlipidemia, we believe that they can also promote implant osseointegration in hyperlipidemia. However, these systems still need further research and confirmation.

For example, simvastatin can also be coated onto the surface of beta-tricalcium phosphate in the form of simvastatin acid to promote bone formation [83,94]. When simvastatin is embedded in polymers, such as PLGA, PEG [84,85,86] and poly(l-lactic acid) (PLA) [87], it is slowly released and promotes osteogenesis. Hydrogels such as gelatins are also widely used carriers for simvastatin [88,89,90,91,92]. Drug-containing nanoparticles are one of the ideal platforms used to deliver simvastatin. They also have excellent osteogenic effects due to their high ratio of surface area to volume, sustained drug release properties, high drug encapsulation rate, enhanced drug permeability, and high stability [85,93]. Overall, the above drug-loading systems can represent ideal materials used to encourage bone formation.

Coating materials have strict requirements for their mechanical and biological properties. The physical properties of coatings are important due to the characteristics of dental implants that are loaded and placed in a long-term lateral force-loading state, and the osseointegration of the implant does not require ingrowth of fibrous tissue between the implant and the bone interface. Therefore, the coating materials used for dental implants under hyperlipidemia should have excellent mechanical properties, a suitable degradation cycle, and the ability to promote osteogenesis.

However, there are still many excellent drug delivery systems that cannot meet the requirements for coating materials. In this situation, using these drug delivery systems for local injection or as a bone grafting material is also a good choice. In general, when the implant is placed, a better initial stability is obtained by making the implant hole smaller than the implant to facilitate osseointegration. Even if the initial stability is excellent, the long-term effect of implantation is still poor in patients with hyperlipidemia due to the decreased osseointegration ability. Therefore, the sacrifice of part of the initial stability and the full embedding of the implant in the bone for a long healing stage, combined with a delayed restoration of the tooth, local injection and added bone grafting materials, can still be applied to dental implants, resulting in a promising effect.

### 6.2. Cerium Oxide Based Implant Modification

When the body is under hyperlipidemia, the body is prone to inflammation due to the high levels of ROS, and the ability of osteoblast differentiation is reduced, seriously affecting the combination between the implant and bone. It would be of great significance to endow the implant surface with an antioxidant function to reduce the level of inflammation around the implant and to improve the activity of osteoblasts around the implant to form a good bone union. A cerium oxide coating has ideal biological properties, since it effectively reduces the content of ROS in osteoblasts under oxidative stress, promotes the proliferation, differentiation and mineralization of osteoblasts, and reduces inflammatory reactions in the body [95]. Therefore, cerium oxide coating is a promising implant modification material under hyperlipidemia.

A cerium oxide nano-catalyzed biological coating has a protective effect on osteoblast activity under oxidative stress and has promoted bone regeneration in animal models of cancellous bone defect. A cerium oxide coating significantly reduces the content of ROS in osteoblasts under oxidative stress and protects the activity and differentiation of osteoblasts from oxidative damage. In addition, a cerium oxide coating significantly reduces the production of oxidizing substances in the tissues around the implants in osteoporotic rats and promotes bone regeneration [96]. It also promotes the proliferation, osteogenic differentiation and mineralization of osteoblasts through the upregulation of BMP and transform growth factors β [97,98]. A high concentration of Ce4+ upregulates the expression of the macrophage anti-inflammatory cytokine interleukin-1 receptor antagonist (IL-1RA), BMP2 and transforming growth factor-1 to reduce the inflammatory reaction and improve osteogenesis. The balance of macrophage anti-inflammatory and pro-inflammatory cytokines is adjusted by the regulation of the concentration of Ce4+, and the anti-inflammatory and bone-promoting microenvironment is created [99]. The new nano-composite material based on mesoporous silica-coated cerium oxide nanoenzyme reduces the circulating level of fatty acids and significantly improves the metabolic phenotype of obese rats. Furthermore, lipomics and gene expression analysis have shown that hyperlipidemia, as well as liver and fat metabolism disorders, were improved [100].

Overall, it can be concluded that cerium oxide is able to improve the adverse effects of implant osseointegration in hyperlipidemia, representing a potential implant modification material that can be applied under hyperlipidemia.

## 7. Discussions

With the development of the economy, obesity has become a major problem in the world [101]. It is often associated with hypertension and hyperlipidemia [102]. Hyperlipidemia has seriously effects on people’s daily lives [103,104,105]. Basic research has proven that hyperlipidemia is harmful to osseointegration, but poor osseointegration does not mean implant failure. Obesity was found to be unrelated to implant failure [106], and a retrospective cohort study revealed that hyperlipidemia had no influence on implants [107]. However, a two-year follow-up study found that hypercholesterolemia was related to peri-implantitis and implant failure (odds ratio = 5.1, P = 0.046) [20]. There are controversial clinical studies on hyperlipidemia; thus, we still need more clinical studies to confirm the effect of hyperlipidemia on osseointegration and implant failure.

At present, the current implant strategies for patients with hyperlipidemia are often based on diet and medication to control the overall blood lipid level of the patient before dental treatment. This process is extremely slow and unstable. Therefore, it is of great significance to study the local modification of implants applied under a high blood lipid environment, which can not only improve the success rate of implant surgery, but also reduce the burden on patients in all aspects. Local drug injection, implant surface modification and bone grafting materials modification are all effective ways to improve the environment around the implant and between the bone.

Simvastatin is a commonly used drug in the treatment of hyperlipidemia [108], and it has the ability to promote osteogenic differentiation. The study of a simvastatin surface modification strategy for implants is essential to evaluate a method for the local delivery of simvastatin. The local application of simvastatin represents an effective method to improve the utilization rate of simvastatin, reduce side effects and promote bone formation, as it has yielded fruitful results. The activation of the Ras-PI3KAkt/MAPK signaling pathway inhibits the formation of cellular cholesterol, has a certain effect in promoting osseointegration, and has certain clinical transformation prospects. Since statins effectively inhibit the differentiation of osteoclasts and reduce the formation of cellular cholesterol, the current modification strategies for implants under hyperlipidemia mainly focus on statins, especially simvastatin. Studies on vitamin D have revealed that hyperlipidemia affects bone metabolism through the mutual regulation of adipocytes, osteoblasts, and osteoclasts, promotes bone loss, inhibits osseointegration, and does not enable the osseointegration of dental implants [109]. Schulze-Spate et al., in a clinical trial in humans, reported that bone remodeling activity was closely related to higher vitamin D levels [109]. Meanwhile, both modification of bone graft material and improvement of implant material are promising strategies for promoting implant osseointegration.

Hyperlipidemia is a lipid metabolism disease affecting bone implant metabolism, depending on the disease status and the complex regulatory mechanism of the body. Understanding the molecular mechanism of lipid metabolism can provide a reference for the development of materials for implant surface modification with the dual function of osteogenesis and lipid reduction.

Because hyperlipidemia increases the risk of periodontal diseases, infection and osteoporosis, it can negatively affect the implant osseointegration process and the success rate of the implant. In addition to implant modification, dentists should arrange necessary preparations before clinical implant surgery for patients with hyperlipidemia. First, it is necessary to minimize the blood lipid level of patients. Second, high bone compression is needed during implant surgery for hyperlipidemic patients. After surgery, patients should take anti-infective drugs in a timely manner to reduce the risk of bacterial infection and should pay close attention to their periodontal health. Further, dentists should delay the loading time of the implant and postpone the appointment time for the second stage of surgery.

In conclusion, the development of more drugs and strategies as well as more in-depth research on the mechanisms of action are urgently needed. Thus, more implant surface modification strategies should be developed in the future to improve the success rate of implant surgery under a hyperlipidemic environment to improve the quality of life of patients with hyperlipidemia.

## Figures and Tables

**Table 1:** Strategies of local drugs injection under hyperlipidemic environment.

No.	Citation	Authors	Carriers	Bioactive Molecules or Drugs	In Vitro	In Vivo	Under Hyperlipidemia Condition
1	[56]	Tan, Jie et al.	No	simvastatin	No	Yes	No
2	[57]	Moriyama, Yasuko et al.	PGA gel	fluvastatin	No	Yes	No
3	[58]	Moriyama, Yasuko et al.	PGA gel	fluvastatin	No	Yes	No
4	[59]	Masuzaki, Tomohiro et al.	PLGA microspheres	fluvastatin	No	Yes	No
5	[60]	Ren, H et al.	No	Sdccag3-enhancer	Yes	Yes	Yes
6	[60]	Ren, H et al.	No	lncRNA-MSTRG.97162.4- enhancer	Yes	Yes	Yes
7	[60]	Ren, H et al.	No	miR-193a-3p-inhibitor	Yes	Yes	Yes
8	[61]	Liu, Fei et al.	No	miR-29a-3p- enhancer	Yes	Yes	Yes

**Table 2:** Promising strategies of implant surface modification under a hyperlipidemic environment.

No.	Citation	Authors	Carriers	Bioactive Molecules or Drugs	In Vitro	In Vivo	Applied to Dental Implant
1	[64]	Nyan, Myat et al.	porous titanium oxide	simvastatin	No	Yes	Yes
2	[65]	Walter, Martin Sebastian et al.	/	simvastatin	No	Yes	Yes
3	[66]	Yang, Guoli et al.	/	simvastatin	No	Yes	Yes
4	[67]	López-Álvarez, Miriam et al.	mesoporous titanium oxide	simvastatin	Yes	No	Yes
5	[68]	Zhao, Shifang et al.	calcium phosphate	simvastatin	Yes	Yes	Yes
6	[69]	Pullisaar, Helen et al.	porous titanium oxide+alginate hydrogel	simvastatin	Yes	No	Yes
7	[70]	Fang, Wen et al.	nanohydroxyapatite	simvastatin	No	Yes	Yes
8	[71]	Salomó-Coll, Oscar et al.	/	vitamin D	No	Yes	Yes

**Table 3:** Promising strategies of statin application in dental implantation.

No.	Citation	Authors	Carriers	Bioactive Molecules or Drugs	In Vitro	In Vivo
1	[83]	Yang, Dae Hyeok, et al.	β-tricalcium phosphate	simvastatin acid	Yes	No
2	[84]	Yan, Qi et al.	PLGA-PEG-PLGA hydrogel	simvastatin	Yes	Yes
3	[85]	Delan, Wisam Khalaf et al.	chitosan+tripolyphosphate nanoparticles	simvastatin	No	Yes
4	[86]	Zhang, Zhan-Zhao et al.	PLGA+collagen	simvastatin	Yes	Yes
5	[87]	Lee, Jung Bok et al.	PLA+gelatin+β-cyclodextrin+hydroxyapatite	simvastatin	Yes	Yes
6	[88]	Park, Yoon Shin et al.	gelatin-PEG-tyramine hydrogel	simvastatin	Yes	No
7	[89]	Tanigo, Tomomi et al.	gelatin+L-lactic acid oligomer	simvastatin	Yes	Yes
8	[90]	Sukul, Mousumi, et al.	gelatin+β-tricalcium phosphate hydrogel	simvastatin	Yes	Yes
9	[91]	Bae, Min Soo et al.	hyaluronic acid hydrogel	simvastatin	Yes	Yes
10	[92]	Zhang, Xiao et al.	LAPONITE^®^ hydrogel	simvastatin	Yes	Yes
11	[93]	Liu, Junjie et al.	calcium–silicon nanospheres	microRNA-210+simvastatin	Yes	Yes

## Data Availability

No new data were created or analyzed in this study. Data sharing is not applicable to this article.
